# Causal Effect of Low‐Density Lipoprotein Cholesterol on Chronic Kidney Disease: A Mendelian Randomization Study

**DOI:** 10.1155/bri/3548900

**Published:** 2026-04-03

**Authors:** Dongming Zhou, Kangchao Zheng, Suwei Zhang, Bingbing Zheng

**Affiliations:** ^1^ Department of Clinical Laboratory, Shantou Central Hospital, Shantou, Guangdong, China, sthospital.com; ^2^ Department of Medical Ultrasonics, Shantou Central Hospital, Shantou, Guangdong, China, sthospital.com; ^3^ Department of Orthopedics, Shantou Central Hospital, Shantou, Guangdong, China, sthospital.com

**Keywords:** chronic kidney disease, IEU Open GWAS, low-density lipoprotein cholesterol, Mendelian randomization, UK Biobank

## Abstract

**Background:**

While dyslipidemia is associated with chronic kidney disease (CKD), conventional observational studies cannot establish causality, and previous Mendelian randomization (MR) findings on low‐density lipoprotein cholesterol (LDL‐C) and CKD remain inconsistent.

**Objective:**

To further investigate the causal relationship between LDL‐C and CKD using MR and evaluate its independence.

**Methods:**

We employed a two‐sample and multivariable MR (MVMR) framework. Initially, two‐sample and reverse MR analyses were performed for four lipid traits on CKD using genetic data from the IEU Open GWAS and the UK Biobank separately, with a Bonferroni‐corrected significance threshold of *p* < 0.0125. Subsequently, MVMR was conducted to assess the independent effect of LDL‐C after adjusting for other lipid traits (*p* < 0.05). Finally, an MVMR model incorporating LDL‐C, smoking, alcohol consumption, and body mass index (BMI) was fitted to test the independence from these lifestyle confounders, followed by a sensitivity analysis with linkage disequilibrium–based confounder filtering to verify robustness.

**Results:**

Two‐sample MR showed a significant causal effect of LDL‐C on increased CKD risk (IEU source: OR = 1.13, 95% CI: 1.03–1.23, *p* < 0.01; consistent results from UK Biobank), with no evidence of reverse causation. MVMR confirmed the independence of this association: LDL‐C remained significantly associated with CKD after adjusting for other lipid traits (OR = 1.21, 95% CI: 1.08–1.35, *p* = 0.0007) and after further adjustment for BMI, smoking, and alcohol (OR = 1.14, 95% CI: 1.04–1.25, *p* = 0.068). The effect direction remained consistent in the stringent sensitivity analysis.

**Conclusion:**

LDL‐C may be an independent risk factor for CKD, the independence of which warrants further validation.

## 1. Introduction

Chronic kidney disease (CKD) represents a significant and growing global health burden, with its prevalence rising particularly rapidly across Asian countries and exhibiting notable regional disparities [1]. It imposes substantial medical and economic costs, elevates risks of cardiovascular mortality and severe infections, and severely impairs patients’ health‐related quality of life [2, 3]. Identifying CKD etiology is crucial for prevention and treatment, and recent advances in genetics and metabolomics have revealed associated genetic variations and altered lipid metabolism, providing new insights into its pathogenesis [4–7].

Although low‐density lipoprotein cholesterol (LDL‐C) is a well‐established causal factor and a primary therapeutic target for cardiovascular disease, its specific causal relationship with CKD remains unclear, and significant discrepancies exist between current clinical guidelines. The Kidney Disease: Improving Global Outcomes guideline notes that in CKD populations, the association between LDL‐C and atherosclerotic risk attenuates as renal function declines [8]. Particularly in dialysis patients, low LDL‐C levels are associated with higher mortality, reflecting confounding by inflammation and malnutrition rather than lower risk. Consequently, LDL‐C alone is not recommended as a criterion for initiating statin therapy in CKD. In contrast, the ACC/AHA guideline classifies CKD as a risk‐enhancing factor for atherosclerotic cardiovascular disease, supporting treatment decisions based on overall cardiovascular risk assessment [9]. Previous research has primarily examined LDL‐C’s role in cardiovascular outcomes among established CKD patients, rather than its potential causal contribution to CKD development [10].

Mendelian randomization (MR), a statistical method that uses genetic variation as an instrumental variable (IV) to assess the causal relationship between exposure factors and diseases, can reduce the impact of confounding factors and reverse causality inherent in traditional observational studies, thereby providing evidence closer to the true causal relationship. The application of the MR method is not limited to the assessment of a single risk factor; it can also consider multiple potential risk factors simultaneously through multivariate MR analysis [11]. The advantage of this approach is that it can more comprehensively evaluate the combined impact of multiple factors on disease outcomes, thereby providing more precise causal inferences. While some MR studies have investigated the lipid–CKD relationship, findings for LDL‐C have been inconsistent [12–14].

To address these gaps, this study employs a systematic, two‐sample MR framework to specifically evaluate the independent causal effect of LDL‐C on CKD risk. We hypothesize that LDL‐C is an independent causal risk factor for CKD. Our analysis will incorporate univariable MR (UVMR), reverse MR, and, most importantly, multivariable MR (MVMR) to isolate the net effect of LDL‐C by adjusting for other lipid traits (HDL‐C, TG, and Lp(a)) and key metabolic confounders (body mass index [BMI], smoking, and alcohol consumption). This study aims to provide robust evidence on the independent etiological role of LDL‐C in CKD.

## 2. Materials and Methods

### 2.1. Study Design

**FIGURE 1:**
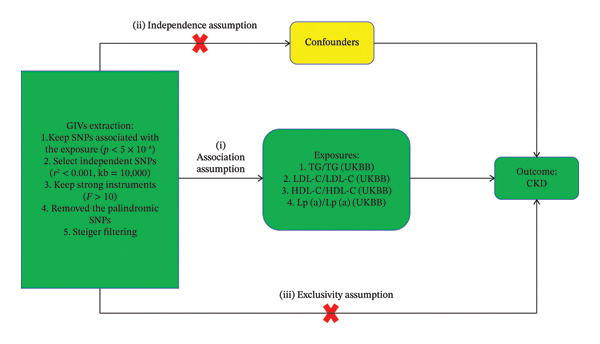
Flowchart of two‐sample Mendelian randomization and three fundamental assumptions of Mendelian randomization. Note: GIVs, genetic instrumental variables; CKD, chronic kidney disease; TG, GWAS dataset of triglycerides from IEU Open GWAS; TG(UKBB), GWAS dataset of triglycerides from UK Biobank; LDL‐C, GWAS dataset of low‐density lipoprotein cholesterol from IEU Open GWAS; LDL‐C(UKBB), GWAS dataset of low‐density lipoprotein cholesterol from UK Biobank; HDL‐C, GWAS dataset of high‐density lipoprotein cholesterol from IEU Open GWAS; HDL‐C(UKBB), GWAS dataset of high‐density lipoprotein cholesterol from UK Biobank; Lp(a), GWAS dataset of lipoprotein(a) levels from IEU Open GWAS; Lp(a) (UKBB), GWAS dataset of lipoprotein(a) levels from the UK Biobank; SNP, single nucleotide polymorphisms.

**FIGURE 2:**
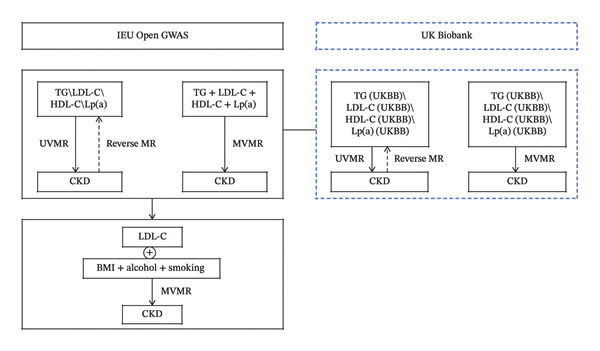
MR analysis of the study design. Note: CKD, chronic kidney disease; TG, GWAS dataset of triglycerides from IEU Open GWAS; TG (UKBB), GWAS dataset of triglycerides from the UK Biobank; LDL‐C, GWAS dataset of low‐density lipoprotein cholesterol from IEU Open GWAS; LDL‐C (UKBB), GWAS dataset of low‐density lipoprotein cholesterol from UK Biobank; HDL‐C, GWAS dataset of high‐density lipoprotein cholesterol from IEU Open GWAS; HDL‐C (UKBB), GWAS dataset of high‐density lipoprotein cholesterol from the UK Biobank; Lp(a), GWAS dataset of lipoprotein(a) levels from IEU Open GWAS; Lp(a) (UKBB), GWAS dataset of lipoprotein(a) levels from the UK Biobank; BMI, body mass index; UVMR, univariable Mendelian randomization; MVMR, multivariable Mendelian randomization.

The study was conducted following a two‐sample MR design (Figures 1 and 2). We initially performed two‐sample UVMR analyses using genetic data from European‐ancestry genome‐wide association studies (GWAS) obtained from the IEU Open GWAS project and the UK Biobank (UKBB). These analyses aimed to evaluate potential causal effects of four lipid traits—LDL‐C, high‐density lipoprotein cholesterol (HDL‐C), triglycerides (TGs), and lipoprotein(a) [Lp(a)]—on CKD. Results indicated a significant causal effect only for LDL‐C on CKD. Reverse MR analysis revealed no evidence of reverse causality for any lipid trait. Subsequently, we conducted MVMR analysis simultaneously incorporating all four lipid traits, which confirmed the significant association between LDL‐C and CKD. Furthermore, an additional MVMR model adjusted LDL‐C for cigarette consumption, alcohol consumption, and BMI—with genetic instruments for these covariates sourced from the IEU Open GWAS database—to test independence from these established lifestyle and metabolic factors. Smoking, alcohol intake, and BMI were selected as covariates because they are well‐established traditional risk factors for both CKD and lipid profiles, consistently identified in numerous epidemiological studies and clinical practice. Genetic instrument selection for all analyses adhered to stringent criteria, including Steiger filtering to ensure correct causal direction. To evaluate robustness, a sensitivity analysis employing linkage disequilibrium (LD)–based confounder filtering was also performed.

### 2.2. Data Sources

To investigate the causal roles of lipid traits in CKD, we obtained genetic association data from large‐scale GWAS conducted exclusively in European‐ancestry populations to minimize population stratification. Exposure data for four lipid traits—LDL‐C, HDL‐C, TGs, and Lp(a)—were sourced from two publicly available databases: the IEU Open GWAS database and the UKBB. For each lipid trait, we utilized summary statistics from independent GWAS published by distinct consortia within these two sources (sample sizes ranged from 115,082 to 441,016 per trait; see Table 1 for consortium and study details). Genetic instruments for lifestyle and metabolic covariates, including cigarette consumption (*n* = 337,334), alcohol consumption (*n* = 335,394), and BMI (*n* = 457,756), were obtained from the IEU Open GWAS database. Outcome data for CKD were derived from the FinnGen database (*n* = 406,745 individuals of European ancestry). All analyses were restricted to European‐ancestry samples to ensure genetic homogeneity.

**TABLE 1:** Information on blood lipids and chronic kidney disease GWAS samples were used in this study.

Trait	GWAS ID	Sample size	Consortium	Populations	Year	PMID
TG	ebi‐a‐GCST90092992	115,082	NA	European	2022	35213538
LDL‐C	ebi‐a‐GCST90018961	343621	NA	European	2021	34594039
HDL‐C	ebi‐a‐GCST90025956	400,754	NA	European	2021	34226706
Lp(a)	ebi‐a‐GCST90025993	348,806	NA	European	2021	34226706
TG (UKBB)	ieu‐b‐111	441016	UK Biobank	European	2020	32203549
LDL‐C (UKBB)	ieu‐b‐110	440546	UK Biobank	European	2020	32203549
HDL‐C (UKBB)	ieu‐b‐109	403943	UK Biobank	European	2020	32203549
Lp(a) (UKBB)	ukb‐d‐30790_irnt	NA	UK Biobank(Neale lab)	European	2018	NA
CKD	finngen_R10_N14_CHRONKIDNEYDIS	406745	—	European	2023	—
Smoke	ieu‐b‐25	337,334	NA	European	2019	30643251
Alcohol	ieu‐b‐73	335,394	NA	European	2019	30643251
BMI	ebi‐a‐GCST90025994	457,756	NA	European	2021	34226706

*Note:* TG, GWAS dataset of triglycerides from IEU Open GWAS; TG (UKBB), GWAS dataset of triglycerides from the UK Biobank; LDL‐C, GWAS dataset of low‐density lipoprotein cholesterol from IEU Open GWAS; LDL‐C (UKBB), GWAS dataset of low‐density lipoprotein cholesterol from the UK Biobank; HDL‐C, GWAS dataset of high‐density lipoprotein cholesterol from IEU Open GWAS; HDL‐C (UKBB), GWAS dataset of high‐density lipoprotein cholesterol from the UK Biobank; Lp(a), GWAS dataset of lipoprotein(a) levels from IEU Open GWAS; Lp(a) (UKBB), GWAS dataset of lipoprotein(a) levels from the UK Biobank.

### 2.3. Genetic Instrument Selection

MR analysis relies on three key assumptions: (i) The association assumption, where genetic instrumental variables (GIVs) are primarily associated with the exposure of interest; (ii) the independence assumption, where the GIVs are not influenced by confounding factors related to the outcome; (iii) the exclusivity assumption, where the effect of the GIVs on the outcome is mediated solely through the exposure and is not influenced by any other biological mechanisms. We selected independent single‐nucleotide polymorphisms (SNPs) (*r* ^2^ < 0.001, 10,000 kb) in the respective data source GWAS, reaching genome‐wide significance level (*p* < 5 × 10^−8^), to serve as genetic instruments for the 6 kinds of exposures. Subsequently, we removed the palindromic SNPs to ensure that the impact of the SNPs on the exposure corresponds to the same allele as their effect on CKD. Using the formula: *F* = (beta^2^/se^2^) [15], we determined the F‐statistic from the allele effect values and standard errors to identify and exclude weak GIVs, with an F value below 10 suggesting their potential presence.

To uphold the fundamental assumption that GIVs should exclusively influence the outcome through exposure, we applied Steiger filtering to confirm that each SNP has a significant primary association with the exposure rather than the outcome [16]. This process ensures the accurate causal pathway from exposure to outcome. Steiger filtering posits that a legitimate GIV should account for a greater variance in the exposure compared to the outcome. If a GIV fulfills this condition, it is marked as “valid”; if not, it is deemed “invalid.”

### 2.4. Statistical Analysis

UVMR analyses were primarily performed using the inverse variance weighted (IVW) method. A Bonferroni‐corrected significance threshold of *p* < 0.0125 (adjusted for the four lipid traits) was applied to account for multiple testing. Reverse MR analyses were conducted to assess and exclude potential reverse causality. For MVMR, two models were constructed. First, all four lipid traits were included simultaneously to estimate their independent effects, using a nominal significance threshold of *p* < 0.05. Second, a multivariable model adjusting LDL‐C for lifestyle factors—specifically cigarette consumption, alcohol consumption, and BMI—was fitted to test the robustness of associations against confounding by these established behavioral and anthropometric measures. To address potential pleiotropy and unmeasured confounding, sensitivity analyses were performed using the weighted median, MR‐Egger, and MR‐PRESSO methods. Horizontal pleiotropy was evaluated via the MR‐Egger intercept test. Furthermore, to enhance causal inference, we performed LD‐based confounder filtering via the LDtrait tool (GRCh37 reference). SNPs in LD (*r* ^2^ ≥ 0.001) with any predefined CKD risk factor were excluded. These factors encompassed: (1) blood pressure traits; (2) glycemic traits; (3) lipid‐lowering interventions, including statin use and PCSK9 levels; (4) fundamental lipid measures, including HDL‐C, TGs, and Lp(a); and (5) traditional renal risk factors, namely, BMI, smoking, and alcohol consumption. Heterogeneity across SNP estimates was assessed using Cochran’s *Q* statistic, with random‐effects IVW models applied when *p* < 0.05. All analyses were conducted in R Version 4.3.2 using the TwoSampleMR package.

## 3. Results

In this study, after excluding SNPs with LD and palindromic structures, a total of 55, 132, 310, and 44 SNPs in TG, LDL‐C, HDL‐C, and Lp(a)samples, respectively, were included as GIVs for MR analysis following Steiger filtering. All included SNPs had F‐statistics exceeding the value of 10, which suggests that the potential for weak instrument bias is minimal.

### 3.1. Two‐Sample MR Analysis

The results of the MR analyses, along with the Cochran *Q* test for heterogeneity and the MR‐Egger intercept test for pleiotropy, are presented in Table 2. Genetic predisposition to higher LDL‐C from the IEU Open GWAS database demonstrated a significant positive causal association with CKD. The IVW method supported this association (OR = 1.13, 95%CI: 1.03–1.23, *p* < 0.01), with consistent direction and significance observed in the weighted median (OR = 1.21, 95% CI: 1.08–1.35, *p* < 0.01) and weighted mode (OR = 1.14, 95% CI: 1.03–1.25, *p* = 0.01) methods, all surpassing the prespecified significance threshold (*p* < 0.0125). This indicates LDL‐C is an independent risk factor for CKD. Sensitivity analyses indicated the presence of heterogeneity (Cochran’s *Q* test *p* < 0.01), but no significant horizontal pleiotropy was detected (MR‐Egger intercept test *p* = 0.67), supporting the robustness of the finding.

**TABLE 2:** Summary Mendelian randomization estimates and the results of Cochran’s *Q* test and pleiotropy test.

Exposures	Outcomes	Number of SNPs	MR methods	OR (95%CI)	β	SE	*p*	Cochran’s *Q* test (*p*)	Egger_ intercept (*p*)	Mean/median *F*‐statistic
TG	CKD	55	MR‐Egger	0.92 (0.74–1.15)	−0.08	0.11	0.47		0.47	98.26/55.41
			Weighted median	0.99 (0.84–1.16)	−0.01	0.08	0.91			
			IVW	0.99 (0.87–1.12)	−0.01	0.06	0.83	0.02		
			Simple mode	1.02 (0.75–1.39)	0.02	0.16	0.89			
			Weighted mode	1.00 (0.85–1.19)	0.00	0.09	0.96			

LDL‐C	CKD	132	MR‐Egger	1.14 (1.02–1.28)	0.13	0.06	0.02		0.67	256.65/57.98
			Weighted median	1.21 (1.08–1.35)	0.19	0.06	< 0.01			
			IVW	1.13 (1.03–1.23)	0.12	0.05	< 0.01	< 0.01		
			Simple mode	1.02 (0.75–1.39)	0.02	0.16	0.91			
			Weighted mode	1.14 (1.03–1.25)	0.13	0.05	0.01			

HDL‐C	CKD	310	MR‐Egger	1.05 (0.96–1.15)	0.05	0.04	0.29		0.61	236.18/57.24
			Weighted median	1.02 (0.92–1.12)	0.02	0.05	0.75			
			IVW	1.07 (1.00–1.14)	0.06	0.03	0.05	< 0.01		
			Simple mode	1.07 (0.86–1.34)	0.07	0.11	0.53			
			Weighted mode	1.02 (0.94–1.10)	0.02	0.04	0.70			

Lp(a)	CKD	44	MR‐Egger	0.97 (0.91–1.04)	−0.03	0.03	0.43		0.38	2256.96/32
			Weighted median	0.97 (0.91–1.04)	−0.03	0.04	0.41			
			IVW	0.98 (0.92–1.05)	−0.02	0.03	0.58	0.28		
			Simple mode	1.07 (0.76–1.51)	0.07	0.17	0.69			
			Weighted mode	0.97 (0.91–1.04)	−0.03	0.03	0.47			

TG (UKBB)	CKD	241	MR‐Egger	0.98 (0.85–1.13)	−0.02	0.07	0.75		0.16	126.66/49.43
			Weighted median	0.92 (0.80–1.06)	−0.09	0.07	0.23			
			IVW	0.90 (0.82–0.98)	−0.11	0.05	0.02	0.04		
			Simple mode	1.16 (0.85–1.60)	0.15	0.16	0.35			
			Weighted mode	0.94 (0.82–1.08)	−0.06	0.07	0.38			

LDL‐C (UKBB)	CKD	142	MR‐Egger	1.12 (0.98–1.28)	0.11	0.07	0.09		0.53	264.78/56.15
			Weighted median	1.21 (1.08–1.35)	0.19	0.06	< 0.01			
			IVW	1.15 (1.04–1.27)	0.14	0.05	< 0.01	< 0.01		
			Simple mode	1.02 (0.76–1.36)	0.02	0.15	0.89			
			Weighted mode	1.15 (1.05–1.26)	0.14	0.05	< 0.01			

HDL‐C (UKBB)	CKD	282	MR‐Egger	1.02 (0.91–1.14)	0.02	0.06	0.74		< 0.01	172.54/53.88
			Weighted median	1.02 (0.89–1.16)	0.02	0.07	0.80			
			IVW	1.14 (1.05–1.24)	0.13	0.04	< 0.01	< 0.01		
			Simple mode	1.24 (0.92–1.68)	0.22	0.15	0.15			
			Weighted mode	1.05 (0.95–1.16)	0.05	0.05	0.37			

Lp(a) (UKBB)	CKD	21	MR‐Egger	0.97 (0.92–1.03)	−0.03	0.03	0.34		0.76	6290.70/784.77
			Weighted median	0.98 (0.94–1.02)	−0.02	0.02	0.29			
			IVW	0.97 (0.92–1.01)	−0.03	0.02	0.14	0.07		
			Simple mode	0.88 (0.79–0.98)	−0.12	0.06	0.04			
			Weighted mode	0.97 (0.93–1.01)	−0.03	0.02	0.12			

*Note:* TG, GWAS dataset of triglycerides from IEU Open GWAS; TG(UKBB), GWAS dataset of triglycerides from the UK Biobank; LDL‐C, GWAS dataset of low‐density lipoprotein cholesterol from IEU Open GWAS; LDL‐C (UKBB), GWAS dataset of low‐density lipoprotein cholesterol from the UK Biobank; HDL‐C, GWAS dataset of high‐density lipoprotein cholesterol from IEU Open GWAS; HDL‐C (UKBB), GWAS dataset of high‐density lipoprotein cholesterol from the UK Biobank; Lp(a), GWAS dataset of lipoprotein(a) levels from IEU Open GWAS; Lp(a) (UKBB), GWAS dataset of lipoprotein(a) levels from the UK Biobank.

Abbreviations: CKD, chronic kidney disease; IVW, inverse variance weighting.

Similarly, LDL‐C derived from the UKBB dataset also showed a significant positive causal relationship with CKD (IVW OR = 1.15, 95% CI: 1.04–1.27, *p* < 0.01). While heterogeneity was noted (Cochran’s *Q* test *p* < 0.01), the MR‐Egger intercept test revealed no evidence of directional pleiotropy (*p* = 0.53).

For HDL‐C from the UKBB dataset (HDL‐C (UKBB)), the IVW method suggested a positive association with CKD (OR = 1.14, 95% CI: 1.05–1.24, *p* < 0.01), meeting the significance threshold. However, other MR methods (MR‐Egger, weighted median, weighted mode) yielded nonsignificant results. Furthermore, the significant MR‐Egger intercept (*p* < 0.01) indicated potential horizontal pleiotropy, suggesting this specific finding regarding HDL‐C (UKBB) may be unreliable. This inconsistency is further supported by the nonsignificant association observed for HDL‐C from the IEU Open GWAS database, reinforcing the likelihood that HDL‐C lacks a significant causal relationship with CKD. Other exposures, including TG and Lp(a), whether assessed using IEU Open GWAS or UKBB data, did not demonstrate statistically significant associations with CKD via the IVW method at the stringent threshold (*p* < 0.0125), indicating a lack of robust causal evidence.

### 3.2. Reverse MR Analysis

Using GIVs for CKD, reverse MR analysis found no statistically significant causal effects of CKD on LDL‐C, HDL‐C, TG, or Lp(a) after Bonferroni correction (*p* < 0.0125). Analyses in both the IEU Open GWAS and UKBB datasets yielded concordant results (all *p* > 0.0125).

### 3.3. MVMR Analysis

**FIGURE 3:**
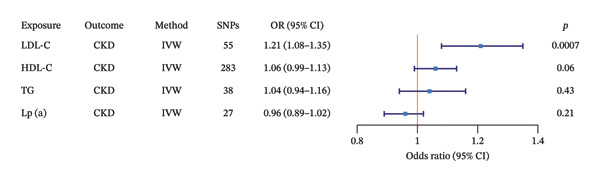
Multivariable MR analysis of LDL‐C, HDL‐C, TG, and Lp(a) on CKD. Note: CKD, chronic kidney disease; TG, GWAS dataset of triglycerides from IEU Open GWAS; LDL‐C, GWAS dataset of low‐density lipoprotein cholesterol from IEU Open GWAS; HDL‐C, GWAS dataset of high‐density lipoprotein cholesterol from IEU Open GWAS; Lp(a), GWAS dataset of lipoprotein(a) levels from IEU Open GWAS; SNPs, single nucleotide polymorphisms; OR, odds ratio; CI, confidence interval.

**FIGURE 4:**
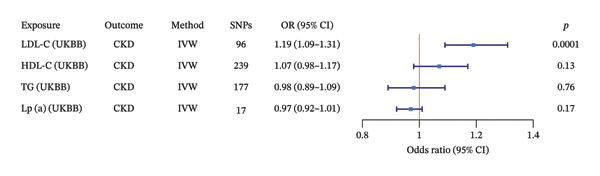
Multivariable MR analysis of LDL‐C (UKBB), HDL‐C (UKBB), TG (UKBB), and Lp(a) (UKBB) on CKD. Note: CKD, chronic kidney disease; TG (UKBB), GWAS dataset of triglycerides from the UK Biobank; LDL‐C (UKBB), GWAS dataset of low‐density lipoprotein cholesterol from the UK Biobank; HDL‐C (UKBB), GWAS dataset of high‐density lipoprotein cholesterol from the UK Biobank; Lp(a) (UKBB), GWAS dataset of lipoprotein(a) levels from the UK Biobank; SNPs, single nucleotide polymorphisms; IVW, inverse variance weighting; OR, odds ratio; CI, confidence interval.

Based on MVMR analyses from two independent genetic databases (IEU Open GWAS and UKBB), we systematically evaluated the independent causal effects of LDL‐C, HDL‐C, TGs, and Lp(a) on the risk of CKD. As shown in Figure 3 (based on IEU Open GWAS data) and Figure 4 (based on UKBB data), the IVW method was used, with a statistical significance threshold set at *p* < 0.05. The results were highly consistent across both datasets: Genetically predicted higher levels of LDL‐C were independently and positively associated with an increased risk of CKD (IEU: OR = 1.21, 95% CI: 1.08–1.35, *p* = 0.0007; UKBB: OR = 1.19, 95% CI: 1.09–1.31, *p* = 0.0001). In contrast, genetically predicted levels of HDL‐C (IEU: OR = 1.06, 95% CI: 0.99–1.13, *p* = 0.06; UKBB: OR = 1.07, 95% CI: 0.98–1.17, *p* = 0.13), TGs (IEU: OR = 1.04, 95% CI: 0.94–1.16, *p* = 0.43; UKBB: OR = 0.98, 95% CI: 0.89–1.09, *p* = 0.76), and Lp(a) (IEU: OR = 0.96, 95% CI: 0.89–1.02, *p* = 0.21; UKBB: OR = 0.97, 95% CI: 0.92–1.01, *p* = 0.17) showed no statistically significant independent causal association with CKD risk.

**FIGURE 5:**
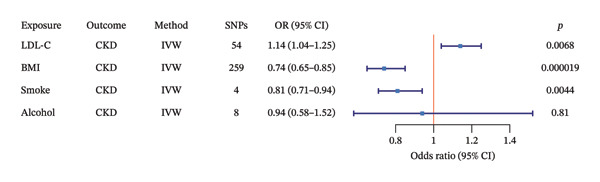
Multivariable MR analysis of the effect of LDL‐C on CKD, adjusted for BMI, smoking, and alcohol. CKD, chronic kidney disease; LDL‐C, GWAS dataset of low‐density lipoprotein cholesterol from IEU Open GWAS; BMI, body mass index; SNPs, single nucleotide polymorphisms; IVW, inverse variance weighting; OR, odds ratio; CI, confidence interval.

When conducting MVMR analysis, we delved deeper into exposure factors that had shown significance in the initial two‐sample MR analysis. After adjusting for BMI, cigarette consumption, and alcohol consumption, LDL‐C still showed a significant statistical association with CKD (OR = 1.14, 95% CI = 1.04–1.25, *p* = 0.0068). The associations between LDL‐C\BMI\cigarette consumption\alcohol consumption and CKD are shown in Figure 5. To rigorously control for potential confounding, a sensitivity analysis with precision confounder filtering based on LD was performed. Under this stringent model (final GIVs comprised 25 SNPs), the MR estimate for the effect of LDL‐C on CKD risk was OR = 1.52, 95% CI = 0.99–2.32, *p* = 0.05. The direction of this effect was consistent with the primary analysis, supporting the robustness of the finding that LDL‐C is an independent risk factor for CKD. Detailed information on the 25 SNPs used in this analysis is provided in Supporting Table 1.

## 4. Discussion

This study robustly established LDL‐C as an independent causal risk factor for CKD. Consistent evidence from MR analyses across two independent datasets confirmed this causal relationship, independent of other lipid fractions. Crucially, MVMR analyses demonstrated that LDL‐C’s causal effect persisted after rigorous adjustment for traditional CKD risk factors (e. g., smoking, alcohol, BMI). Even under extremely stringent sensitivity analyses with LD‐confounder‐filtered IVs, the effect direction of LDL‐C on CKD risk remained unified with all primary findings. The high concordance across complementary analytical approaches solidly supports the robustness of LDL‐C’s independent causal role in CKD pathogenesis.

Previous MR studies investigating the causal relationship between LDL‐C and CKD have yielded inconsistent conclusions. First, key methodological variations in prior research have led to conflicting results. ① Inadequate disentanglement of interrelationships among lipid components: Many studies employed only UVMR, whose estimated “total effect” may be confounded by the mediating or confounding effects of other correlated lipids (e.g., HDL‐C, TG). For instance, while a prospective study by Wang et al. observed a phenotypic association between LDL‐C and CKD, their UVMR analysis found no causal effect, suggesting that the initial observational link may be susceptible to confounding [17]. Similarly, Rasheed et al. demonstrated that the effect of LDL‐C on kidney function was attenuated after adjusting for other lipids in MVMR, indicating that the total effect estimated in univariable analyses can be influenced by lipid correlations [18]. ② Potential horizontal pleiotropy of IVs: If the selection of IVs fails to sufficiently exclude SNPs genetically associated with known CKD risk factors (e.g., blood pressure, blood glucose, BMI), estimation bias may occur. A comprehensive trans‐ethnic MR study by Zheng et al. highlighted the importance of addressing pleiotropy, showing that after rigorous IV selection and sensitivity analyses, the causal estimates for several cardiometabolic risk factors were refined [19]. ③ Differences in study populations and statistical power: Variations in genetic backgrounds, CKD definitions, and sample sizes across different ethnic populations (European, East Asian, African ancestry) may affect the reproducibility of results. For example, a study in an African‐ancestry population by Kintu et al. reported that genetically predicted higher LDL‐C was associated with higher eGFR, a finding that contrasts with some studies in European populations, suggesting potential ancestral differences in the genetic architecture of lipids and kidney function [20]. Furthermore, an analysis by Emanuelsson et al., while confirming a causal role for LDL‐C in peripheral arterial disease, showed no such relationship with CKD in European cohorts, underscoring that findings can be outcome‐specific and population‐dependent [14].

Second, the design of this study systematically addresses the aforementioned issues. ① Application of MVMR to isolate independent effects: We not only performed UVMR analysis but, more crucially, conducted MVMR analysis simultaneously incorporating LDL‐C, HDL‐C, TG, and Lp(a) into the model. The results consistently showed that only LDL‐C retained independent significance, thereby demonstrating its effect independent of other major lipids. ② Implementation of an unprecedentedly stringent IV selection to minimize pleiotropy: We additionally applied LD‐based precise confounder filtering (using the LDtrait tool) to systematically exclude SNPs in LD with a predefined set of potential confounders, including blood pressure, blood glucose, statin use, PCSK9 levels, other lipids, BMI, smoking, and alcohol consumption. This near‐“overcontrolled” screening maximally reduces bias from genetic pleiotropy and ensures the specificity of the IVs. ③ Replication using different data sources: Our analyses utilized exposure data from both the IEU Open GWAS and the UKBB, and consistent conclusions were obtained across these two independent datasets, enhancing the reliability of the findings. ④ Exploration and acknowledgment of potential nonlinear relationships: We noted that the study by Wang et al. suggested a possible nonlinear association between LDL‐C and CKD (e.g., increased risk when LDL‐C is below 3.5 mmol/L) [21]. Our linear analyses provide a robust foundation for such future investigations.

The causal effect of LDL‐C on CKD inferred from our genetic analysis is underpinned by several well‐established biological pathways. The observed association is mechanistically plausible, potentially mediated through the following processes: First, under the oxidative stress milieu common in CKD, LDL‐C is readily oxidized to form oxidized LDL (ox‐LDL). Uptake of ox‐LDL by renal cells promotes foam cell formation and glomerular injury [22, 23]. Second, ox‐LDL activates proinflammatory signaling, upregulating adhesion molecules and chemokines, which recruit monocytes and exacerbate renal inflammation and fibrosis [23, 24]. Furthermore, LDL‐C accumulation can induce lipotoxicity, disrupting cellular lipid homeostasis via SREBP activation and impairing cholesterol efflux, leading to endoplasmic reticulum stress and apoptosis in pivotal renal cells [24]. Collectively, these synergistic processes of oxidative stress, inflammation, and lipotoxicity provide a coherent pathological framework that aligns with and biologically validates our genetic findings [25]. Building on these pathways, our findings encourage further investigation into specific cellular mechanisms and upstream regulators. The genetic variants used as IVs for LDL‐C likely reflect lifelong alterations in lipid metabolism genes (e.g., LDLR, PCSK9), potentially with tissue‐specific effects. We hypothesize that genetically elevated LDL‐C may disproportionately affect renal podocytes and proximal tubular epithelial cells, which are highly vulnerable to lipotoxicity due to their limited regenerative capacity and substantial metabolic demands. Future studies should utilize single‐cell transcriptomics on renal tissues from individuals with high LDL‐C genetic risk scores to elucidate cell‐type‐specific responses—such as NLRP3 inflammasome activation. Such approaches will bridge population‐genetic insights with precise molecular mechanisms underlying the LDL‐C–CKD axis.

Our study, employing rigorous two‐sample and MVMR analyses, revealed a significant genetic causal relationship between LDL‐C and CKD, but not for TGs, HDL‐C, or Lp(a). This discrepancy may be attributed to several factors. Methodologically, the stringent Bonferroni‐corrected significance threshold (*p* < 0.0125) applied in univariable analyses, while controlling for Type I error, might have reduced the statistical power to detect weak to moderate genetic effects. Biologically, the pathogenic pathways of different lipid fractions likely exhibit specificity: LDL‐C primarily drives CKD progression through mechanisms such as promoting atherosclerosis, intraglomerular lipid deposition, and direct renal cell toxicity. In contrast, the protective effect of HDL‐C, the association of TG, or the proinflammatory/prothrombotic effects of Lp(a) might play a relatively minor role in CKD pathogenesis or be masked by the dominant effect of LDL‐C, a notion further supported by MVMR analyses where only LDL‐C remained significant under a more lenient threshold. Furthermore, the robustness of the causal inference for LDL‐C was strengthened by reverse MR, multivariable adjustments, and sensitivity analyses controlling for known confounders (e.g., BMI, smoking, alcohol consumption), suggesting that the causal relationship for other lipids might indeed be weak. These findings have important implications for future research, which should focus on investigating finer lipid subfractions (e.g., LDL particle density), exploring potential nonlinear relationships, and replicating these findings in larger and more diverse populations to fully elucidate the causal network of dyslipidemia in CKD.

Despite the strengths of our methodological approach, several limitations warrant consideration. First, the predominantly European ancestry of our sample limits the generalizability of our findings to other populations. Genetic architectures and lifestyle factors can vary substantially across ancestries, and future research should prioritize diversifying sample populations. Second, although we employed rigorous criteria for genetic instrument selection (e.g., F‐statistic > 10, Steiger filtering) to minimize weak instrument bias and ensure correct causal direction [26, 27], these methods may not entirely eliminate potential pleiotropy or exclude all valid instruments. Future studies could benefit from more sophisticated methods to explore and account for horizontal pleiotropy. Finally, while we adjusted for several key confounders, unmeasured environmental factors or complex gene–environment interactions could still influence the risk of CKD. Future investigations incorporating richer environmental data are needed to elucidate these potential interactions more fully. Further studies are needed to determine whether interventions targeting LDL‐C could translate into clinical benefits for CKD prevention. It is important to note that our study establishes an etiological link but does not directly quantify the absolute risk increase in clinical settings or the potential public health impact of LDL‐C lowering. Translating this genetic evidence into clinical practice requires further evidence from prospective observational studies and randomized controlled trials to estimate effect sizes, evaluate cost‐effectiveness, and define optimal treatment thresholds in diverse CKD populations.

## 5. Conclusions

This etiological investigation provides compelling evidence that genetically elevated LDL‐C is an independent causal factor for CKD, distinct from conventional lipid traits and lifestyle confounders. These findings establish LDL‐C as a primary etiological driver in renal pathology, suggesting lipid‐specific pathways as targets for nephroprotective interventions.

**Nomenclature**
CKD: Chronic kidney disease
GIVs: Genetic instrumental variables
GWAS: Genome‐wide association studies
SNP: Single‐nucleotide polymorphism
MR: Mendelian randomization
LDL‐C: Low‐density lipoprotein cholesterol
HDL‐C: High‐density lipoprotein cholesterol
BMI: Body mass index
UKBB: UK Biobank
UVMR: Univariable Mendelian randomization
MVMR: Multivariable Mendelian randomization
TG: Triglycerides
LP(a): Lipoprotein(a)
IVW: Inverse variance weighting
OR: Odds ratio

## Author Contributions

Dongming Zhou and Kangchao Zheng performed the data analyses and wrote the manuscript. Suwei Zhang analyzed the data and revised the manuscript. Bingbing Zheng participated in the study design and helped draft the manuscript.

## Funding

No funding was received for this research.

## Ethics Statement

We utilized publicly available GWAS summary statistics. Data for the outcome (CKD) came from the FinnGen study (https://www.finngen.fi/en). Data for all exposures and covariates were extracted from the IEU Open GWAS platform (https://gwas.mrcieu.ac.uk), which consolidates data from multiple sources, including the UK Biobank. The data sources of this study are based on secondary analysis of previously published GWAS, so informed consent and ethical approval can be detailed in the original studies.

## Conflicts of Interest

The authors declare no conflicts of interest.

## Supporting Information

Additional supporting information can be found online in the Supporting Information section.

## Supporting information


**Supporting Information 1** Supporting Table 1. Characteristics of the 25 genetic instrumental variables used as instruments for LDL‐C in the stringent linkage disequilibrium‐based sensitivity analysis for CKD risk.

**Supporting Information 2** Supporting Table 2. Sensitivity analysis results examining the effect of palindromic SNP handling (using action = 2 in the harmonization procedure) to ensure robustness against strand ambiguity.

**Supporting Information 3** Supporting Table 3: Comprehensive instrument details for all lipid traits. This file contains eight worksheets with data for TG, TG (UKBB), LDL‐C, LDL‐C (UKBB), HDL‐C, HDL‐C (UKBB), Lp(a), and Lp(a) (UKBB). Each worksheet provides SNP identifiers, effect alleles, F‐statistics, and variance explained.

## Data Availability

The datasets discussed in this research are available in online databases. You can find the names of these databases and their respective accession numbers within the article itself.
